# Quality assurance and field characterisation for MRgHIFU treatments: their need and the challenges presented

**DOI:** 10.1186/2050-5736-3-S1-O66

**Published:** 2015-06-30

**Authors:** Gail ter Haar, Ian Rivens, John Civale, Chris Bunton, Richard Symonds-Tayler

**Affiliations:** 1 The Institute of Cancer Research, London, United Kingdom

## Background/introduction

In our drive to increase the clinical recognition of HIFU treatments, it is important that we pay attention to other comparable, but more widely accepted, therapeutic techniques, and match their rigorous quality assurance and calibration practices.

Well validated Quality Assurance (QA) and field characterisation techniques are important in order that treatments can be planned and simulated, and so they may be compared between patients, between centres and between machines. There is still some discussion as to which the most relevant parameters for such comparisons are.

While the pressure distribution and total power can be measured with reasonable accuracy in the laboratory, the presence of the high magnetic fields in the vicinity of an MR scanner render many of the current equipment unuseable. It is therefore important to develop techniques appropriate for MRgHIFU systems that work within the restricted space available.

## Methods

We are building an MR compatible acoustic power measurement system which is designed to allow measurements to be made in the magnet bore in a similar way to those made in the laboratory. This system consists of a castor oil target immersed in water, and is connected by fishing wire to a load cell which enables measurement of the weight of the buoy. The load cell has been calibrated and allows measurement of the ultrasonic radiation force and buoyancy force due to thermal expansion of the castor oil. The use of a load cell instead of the dedicated laboratory balance presents problems in terms of oscillations in the measured weight due to the rapid onset/offset of the acoustic radiation force. Comparative measurements have been made in the laboratory, using a non-MR compatible target, a Sartorius balance and the load cell. We have also designed and built a positioning system which uses electric motors that make use of the static magnetic field to operate. This system allows positioning of a hydrophone (Onda HGL-0200) as pressure field mapping.

## Results and conclusions

Laboratory testing of the load cell *vs*. the Sartorius balance showed both that oscillations in the measured weight using the load cell could be successfully removed by applying a carefully chosen time averaging window and that there was good agreement between the acoustic power measured with the Sartorius and the load cell. Our next steps are to build an MR compatible target, a water tank that fits into the bore of the magnet and provides coupling to a Sonalleve HIFU transducer and then to verify use of the system in the magnet. The MR compatible positioning system allows precise (sub millimetre) location of the focal peak that is not achievable using the MR positioning system. The output from the Onda hydrophone could be read once it had been passed through a coax connector grounded to the scanners RF cage. Currently software is being written for automated beam plotting in the bore of the MR scanner. Results will be presented at the meeting. Pressure distributions using the modified gantry and MR compatible pressure sensors will also be presented. Fields mapped in the magnet bore will be compared with those obtained in the laboratory for our MR compatible transducers.

**Figure 1:**
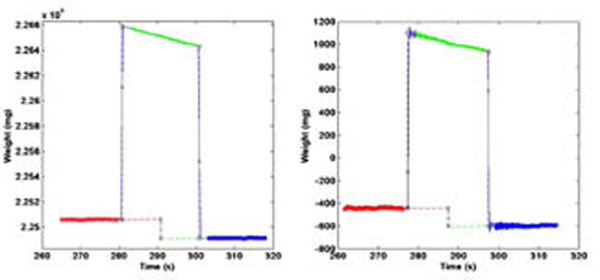
Test sonication with the Sartorius balance (left), and load cell (right). Note the increased oscillations with the load cell, e.g., just before 280 seconds.

